# ECMO for Metabolic Crisis in a Patient with Mitochondrial Disease

**DOI:** 10.1155/2021/9914311

**Published:** 2021-11-01

**Authors:** Sonal Sharma, Clifford Deerman, Michael H. Andreae, Conrad Myler

**Affiliations:** Department of Anesthesiology and Perioperative Medicine, Penn State Health, Milton S. Hershey Medical Center, Hershey, PA 17033, USA

## Abstract

Patients with mitochondrial disease exhibit disrupted pyruvate oxidation, resulting in intraoperative and perioperative physiologic derangements. Increased enzymatic conversion of pyruvate via lactate dehydrogenase during periods of fasting or stress can lead to metabolic decompensation, with rapid development of fatal lactic acidosis. We describe the intraoperative management and postoperative critical care of a patient with mitochondrial disease who presented for repair of esophageal perforation following repair of a paraesophageal hernia. His surgery was complicated by the development of metabolic crisis and severe lactic acidosis which became resistant to conventional therapy before ultimately resolving with the initiation of venoarterial extracorporeal membrane oxygenation (VA-ECMO).

## 1. Introduction

Mitochondrial disease, once thought to be rare, is now recognized as fairly common, with an incidence of about 1 per 4–5,000 live births [1]. Disruption of the mitochondrial pyruvate oxidation pathway in these patients leads to increased conversion of pyruvate via the enzyme lactate dehydrogenase, which is generally well compensated at baseline without specific therapy. However, during periods of increased metabolic stress such as sepsis or prolonged fasting, patients with mitochondrial disorders have a high risk of metabolic decompensation and severe lactic acidosis. Unfortunately, treatment is limited to supportive care, as disease-specific therapy is not available. Even with aggressive treatment, this condition may be rapidly fatal [2–4].

Venoarterial extracorporeal membrane oxygenation (VA-ECMO) has been successfully used to support diverse patients with cardiorespiratory failure from a variety of medical and surgical conditions [5, 6]. However, we have not found reports utilizing VA-ECMO to support patients with mitochondrial disease in metabolic crisis.

We describe the intraoperative management and postoperative critical care of a young man with mitochondrial disease presenting for esophageal perforation that developed following paraesophageal hernia repair and got further complicated by metabolic crisis and severe metabolic acidosis, which proved resistant to conventional therapy before resolving following initiation of VA-ECMO.

This manuscript adheres to the EQUATOR guidelines. HIPAA authorization to publish this case report was obtained from the patient's family.

## 2. Case Presentation

A 30-year-old, 75 kg, ASA 3, male patient presented for repair of paraesophageal hernia. His medical history was significant for Leigh syndrome due to MRSP34 deficiency which had resulted in progressive neurological deterioration. The neurological symptoms were marked by cognitive dysfunction, tremors, peripheral neuropathy, and balance problems for which he was taking biotin, riboflavin, ubiquinone, vitamin E, and lipoic acid. His baseline venous lactic acid was 2-3 mmol/L. Preoperative arterial blood gas (ABG) showed pH 7.34, partial pressure of carbon dioxide (PaCO_2_) 39.8 mmHg, partial pressure of oxygen (PaO_2_) 97 mmHg, bicarbonate (HCO_3_) 21.9 mmol/L, and base excess −3. Preoperative transthoracic echocardiography (TTE) revealed a left ventricular ejection fraction (LVEF) of 65% and no valvular dysfunction. He underwent a robotic transabdominal paraesophageal hernia repair under general anesthesia, requiring modest doses of norepinephrine and vasopressin. Intraoperatively, he received 3 L of plasmalyte, 750 mL of 5% albumin, and 100 meq sodium bicarbonate. Estimated blood loss (EBL) was <50 mL. Postoperatively, patient was hemodynamically stable, extubated, and was recovering in the intensive care unit (ICU) for close monitoring given the known history of mitochondrial disease.

On postoperative day (POD) 1, a chest X-ray demonstrated a new pneumomediastinum, and he was taken back to the operating room for repair of a suspected esophageal perforation. During the repair of the esophageal perforation, the first intraoperative arterial blood gas (ABG) revealed a pH of 7.17, PaCO_2_ 22.8 mmHg, HCO_3_ 8.3 mmol/L, base deficit 18, and lactate 9.7 mmol/L. Throughout the case, it became increasingly difficult to maintain a mean arterial pressure (MAP) > 65 mmHg despite fluid resuscitation and escalating infusions of norepinephrine and vasopressin. The acidosis also continued to worsen with a pH 7.07. At this point, tromethamine (THAM) infusion was initiated, and 10% dextrose was substituted for plasmalyte as maintenance intravenous fluid. This resulted in an improvement in ABG (pH of 7.2, base deficit of 13 mmol/L, and HCO_3_ of 13 mmol/L), but the lactate level remained the same, and he continued to require significant vasopressor support. He received 5 L of plasmalyte and 750 ml of 5% albumin during the procedure. The EBL was 50 ml with 2.5 L urine output.

The patient was transported intubated and sedated to the surgical intensive care unit where high-volume continuous venovenous hemodiafiltration (CVVHDF) was promptly initiated (effluent dose 35 mL/kg/hr) for acute acidosis despite adequate urine output. He remained hypotensive with MAPs of 45–60 mmHg despite norepinephrine 0.32 mcg/kg/min and vasopressin 0.04 U/min. His acidosis continued to worsen with arterial pH 7.05 and lactate 16 mmol/L. A TTE was done which showed a LVEF of 35%, with diffuse hypokinesis. The right ventricle was mildly dilated, but with normal systolic function. There was moderate to severe tricuspid regurgitation (calculated right ventricular systolic pressure (RSVP) 33.5 mmHg) and mild to moderate mitral regurgitation. An epinephrine infusion was added to improve inotropy.

Unfortunately, at this time, the patient also developed severe hypoxic respiratory failure, with chest X-ray showing bilateral pulmonary infiltrates despite mechanical ventilatory support with synchronized intermittent mandatory ventilation with pressure support with tidal volume 480 ml, positive end expiratory pressure 10, pressure support of 10 cm H_2_O, and fractional inspired concentration of oxygen at 60%. ABG on these settings revealed pH 7.04, PaCO_2_ 28 mmHg, PaO_2_ 67 mmHg, HCO_3_ 7.6 mmol/L, and base deficit of 21.7. Due to persistent hypoxemia, progressive acidosis, and hypotension, the ECMO team was consulted, and they decided to proceed with VA-ECMO. The patient was cannulated with 23 Fr venous and 17 Fr arterial cannulae (Abbott Cardiovascular, Plymouth, Minnesota, USA) in the right femoral vessels and with a 9Fr reperfusion cannula to the superficial femoral artery (Maquet Cardiopulmonary AG, Hirrlingen, Germany). Dextrose infusion was increased to 6 mg/kg/min, and total parenteral nutrition was started. Insulin infusion was used to maintain the blood glucose level in the range of 100–120 mg/dL.

**Figure 1:**
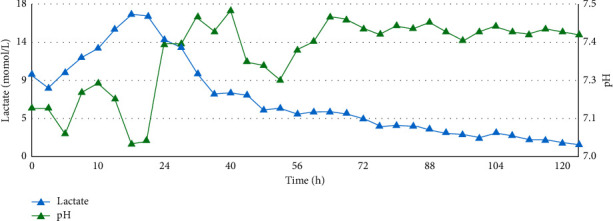
Trend showing lactate and pH values with time.

Within two hours of VA-ECMO initiation, the patient's lactic acidosis improved (Figure 1). By POD 5, his lactate was 1.5 mmol/L, and by POD 6, he no longer required vasopressors, inotropes, or CVVHDF (Table 1). VA-ECMO was discontinued without complications. The patient required tracheostomy due to slowly resolving hypoxemic respiratory failure. TTE on POD 15 revealed normal size and function of all cardiac chambers. LVEF was 70%, with no significant valvular abnormality. He was successfully discharged.

## 3. Discussion

In the present case, we report the treatment of a 30-year-old male with mitochondrial disease and combined distributive and cardiogenic shock using VA-ECMO.

The likely causes of the patient's intraoperative decompensation during the repair of esophageal perforation included a combination of distributive shock secondary to sepsis, vasoplegia, lack of physiologic reserve to meet increased metabolic demands due to mitochondrial dysfunction, and intolerance of acidosis. This resulted in acute cardiomyopathy as was evident in the postoperative TTE. There was concern that his cardiac dysfunction would continue to result in inadequate tissue perfusion and worsening aerobic respiration, further exacerbating his inability to clear metabolites, while his mitochondrial disease with severe acidosis would continue to worsen his cardiac dysfunction in a vicious cycle.

Severe acidosis is known to have deleterious effects on many cellular functions, including those of the cardiovascular system. Increased proton concentrations in blood can lead to decreased cardiac contractility caused by reduced myofilament calcium sensitivity and decreased influx of calcium into myocardial cells with concurrent arterial vasodilation and venous vasoconstriction [7–10]. Acidosis leads to stimulation of the sympathetic nervous system, but the response to catecholamines is decreased [11]. Closure of gap junctions slows the impulse conduction of the heart, leading to a high incidence of dysrhythmias [12].

The initial management of metabolic crisis in patients with mitochondrial disease focuses on fluid and caloric supplementation [12]. Hypoglycemia and catabolism should be avoided as these may contribute to anaerobic respiration and acidemia. An initial glucose infusion rate of 5-6 mg/kg/min, with close monitoring of serum glucose and lactate levels, has been recommended [12]. Serum glucose should not exceed 100–120 mg/dl as excessive glucose supplementation may also worsen lactic acidosis. Simultaneous insulin infusion may be required to maintain serum glucose levels within the recommended range.

There is conflicting evidence on the effects of infusing a buffer solution during metabolic acidosis [13–16]. As severe metabolic acidosis may cause cellular tissue damage, buffer therapy in the form of sodium bicarbonate (NaHCO_3_) or THAM may be used in severe lactic acidosis. However, studies have suggested that it may cause harm by increasing arterial and tissue capillary PaCO_2_ and, thereby, worsen intracellular acidosis [17] which may have occurred in our case.

Renal replacement therapy (RRT) may also be used for the treatment of lactic acidosis [18]. Advantages of using RRT over buffering with alkalinizing substances include prevention of hyperosmolarity and volume overload. This approach can lead to efficient removal of lactate, but a rebound may occur upon cessation of RRT if the cause of excess lactate production has not resolved. Moreover, failure to clear lactate despite adequate dose of RRT, such as in our patient, is possible if the rate of production exceeds the rate of clearance. In our patient, the addition of buffering solutions and RRT did not improve laboratory markers of acidosis or hemodynamic parameters.

VA-ECMO may be a useful treatment modality in reversible cardiogenic shock. While ECMO does not remove or neutralize lactic acid, it can provide improved clearance of carbon dioxide and delivery of oxygenated blood, which may in turn reduce anaerobic hydrolysis. Mechanical circulatory support can avoid secondary injury to other organs due to hypoperfusion while awaiting cardiopulmonary recovery. Our patient displayed signs of severe hemodynamic and metabolic compromise prior to receiving ECMO. Given the trajectory of his clinical deterioration despite maximal medical therapy, he was unlikely to survive without that treatment. The severe metabolic acidosis from his acute on chronic comorbidities resulted in impaired cardiac contractility and hypoperfusion, causing a vicious cycle of worsening acidosis and cardiac dysfunction. The significant and rapid improvement after adequate oxygenation and perfusion with ECMO supports the importance of adequate oxygen delivery in these patients. The optimal time to initiate ECMO therapy remains unclear. Recent recommendations suggest that ECMO should be initiated when the low cardiac output syndrome (cardiac index (CI) < 2 L/min/m^2^) lasts >3 hours and/or is accompanied by metabolic acidosis (base excess > −5 mmol/L). The optimal window is missed if base excess > −5 mmol/L persists for >12 hours and lactic acid level >10 mmol/L persists for >10 hours [19]. In our case, timing of VA-ECMO seemed to be appropriate.

ECMO may be considered as a supportive modality in selected patients with mitochondrial disease suffering from acute potentially reversible metabolic crisis which is resistant to maximal medical therapy.

## Figures and Tables

**Table 1:** Trend of vital signs and dose of vasopressors after initiation of venoarterial extracorporeal membrane oxygenation.

	Heart rate	Blood pressure (mmHg)	Central venous pressure (mmHg)	Epinephrine dose (mcg/kg/min)	Norepinephrine dose (mcg/kg/min)	Vasopressin dose (units/min)
Day 1	127	79/59	12	.12	0.2	0.08
Day 2	93	99/86	12	.14	0.08	0.06
Day 3	88	107/62	7	.08	—	—
Day 4	94	114/67	8	—	—	—

## Data Availability

No data were used to support this study.
